# Real-World Clinical Experience of First-Line Ribociclib Combined with an Aromatase Inhibitor in Metastatic Breast Cancer

**DOI:** 10.3390/cancers18020242

**Published:** 2026-01-13

**Authors:** Ana S. Cvetanović, Kristina B. Jankovic, Ana S. Stojković, Nikola D. Živković, Miloš S. Kostić, Lazar S. Popović

**Affiliations:** 1 Department for Oncology, Medical Faculty Nis, University of Nis, 18000 Nis, Serbia; 2 Clinic of Oncology, University Clinical Centre Nis, 18000 Nis, Serbia; jankovictina11@gmail.com (K.B.J.); anastojka97@gmail.com (A.S.S.); 3 Department for Pathology, Medical Faculty Nis, University of Nis, 18000 Nis, Serbia; nikola.zivkovic@medfak.ni.ac.rs; 4 Center for Pathology, University Clinical Centre Nis, 18000 Nis, Serbia; 5 Department for Immunology, Medical Faculty Nis, University of Nis, 18000 Nis, Serbia; milos.kostic@medfak.ni.ac.rs; 6 Department of Medical Oncology, Oncology Institute of Vojvodina, 21000 Novi Sad, Serbia; lazar.popovic@mf.uns.ac.rs; 7 Faculty of Medicine, University of Novi Sad, 21000 Novi Sad, Serbia

**Keywords:** ribociclib, real world data, progression-free survival, adverse events

## Abstract

Background/Objectives: Despite initial sensitivity to ET, most patients with HR+/HER2− breast cancer develop resistance. A key molecular mechanism of endocrine resistance in HR+ breast cancer involves dysregulation of the cyclin D–CDK4/6–Rb signaling axis, which controls the transition from the G1 to S phase of the cell cycle. Introducing cyclin-dependent kinase 4 and 6 inhibitors (CDK4/6i) has changed therapeutic paradigms in HR+/HER2− breast cancer, as their synergistic use with endocrine therapy significantly prolongs progression-free survival (PFS) and effectively mitigates clinically relevant endocrine resistance in this patient population compared to ET alone. The aim of our study was to evaluate patients’ clinical characteristics, the clinical effectiveness of treatment, measured by progression-free survival (PFS), and the safety profile of combined ribociclib (CDK4/6i) and standard endocrine therapy (aromatase inhibitor) as a first-line treatment for patients with HR+/HER2− advanced or metastatic breast cancer at the Clinic of Oncology, University Clinical Centre Nis, Serbia. Methods: In this study, we present a retrospective prospective analysis of all patients with metastatic HR+/HER2− breast cancer treated with a combination of ribociclib and aromatase inhibitors in the first-line treatment of metastatic HR+/HER2− BC between June 2022 and January 2025, with a follow-up completed in October 2025. A total of 132 patients who met the criteria were included. Results: The median progression-free survival (PFS) in the entire group was 30 months, while the 12-, 24-, and 36-month PFS were 82.15%, 72.24%, and 28.75%, respectively. The overall response rate (ORR) was 41.7%, while the clinical benefit rate (CBR) was 89.3%. There was no statistically significant difference in PFS with respect to tumor grade (*p* = 0.54), Ki 67 level (<20% vs. >20%, *p* = 0.83), or the type of adjuvant endocrine therapy used (tamoxifen vs. AI) It is important to emphasize that female patients who had not previously received chemotherapy had a better response to ribociclib compared to those who had (33 m vs. 28 m, *p* = 0.05). Although a numerical difference in PFS was found in patients with bone-only metastases compared to those with metastases in other organs, the difference was not statistically significant (PFS 33 m vs. 30 m, *p* = 0.27;), and efficacy was consistent across menopausal status groups. The most common adverse effect was neutropenia, occurring in 89.4% of patients, 47.7% of whom presented with grade 3 or 4. As for hepatotoxicity, transaminase increase occurred in 25 patients (18.8%), 5 of whom (3.8%) were grade 3–4, and QTc interval prolongation occurred in 5.3% of patients. Conclusions: The results in terms of PFS and AEs are consistent with those of pivotal studies and real clinical practice data, but a direct comparison is not possible due to differences in patient populations. Ribociclib once again demonstrated efficacy in all patient subgroups and remains the gold standard, alongside ET, for first-line HR+/HER2-negative mBC.

## Simple Summary

Introducing cyclin-dependent kinase 4 and 6 inhibitors (CDK4/6i) has changed therapeutic paradigms in HR+/HER2− breast cancer, as their synergistic use with endocrine therapy significantly prolongs progression-free survival (PFS) and effectively mitigates clinically relevant endocrine resistance in this patient population compared to ET alone. Our study included 132 patients and aimed to evaluate the clinical characteristics of patients, the clinical effectiveness of treatments measured by progression-free survival (PFS), and the safety profile of combined ribociclib (CDK4/6i) and standard endocrine therapy (aromatase inhibitor) as a first-line treatment for patients with HR+/HER2− advanced or metastatic breast cancer in our center. The median progression-free survival (PFS) across the entire group was 30 months, while the 12-, 24-, and 36-month PFS values were 82.15%, 72.24%, and 28.75%, respectively. The overall response rate (ORR) was 41.7%, while the clinical benefit rate (CBR) was 89.3%. In terms of PFS and AEs, the results are consistent with pivotal studies and real clinical practice data, but a direct comparison is not possible due to differences in patient populations.

## 1. Introduction

Breast cancer is a leading global health problem with the highest incidence rate amongst malignant diseases. In 2022 alone, about 2.3 million new cases of breast cancer were registered, making it the most frequently occurring malignancy amongst women globally; furthermore, breast cancer is the leading cause of cancer-related mortality, with an estimated 670,000 deaths in the same year [1]. Hormone-receptor-positive and HER2-negative (HR+/HER2−) breast cancer is the most common molecular subtype, comprising about 75% of all diagnosed cases [2]. It is characterized by estrogen (ER) and/or progesterone receptor (PR) expression, with human epidermal growth factor 2 (HER2) amplification or overexpression absent [3]. Due to the dependence of tumor cells on estrogenic signals for growth and proliferation, endocrine therapy (ET) remains the backbone of both adjuvant management and metastatic stage treatment of the disease in this patient population.

Despite initial sensitivity to ET, most patients with HR+/HER2− breast cancer develop resistance, whether primary (de novo), which is present in approximately 10–20% of patients, or secondary, manifested during prolonged exposure to hormone therapy [4]. Endocrine resistance mechanisms are multifaceted and include complex molecular alterations and alternative signaling pathway activation, which allow tumor cells to overcome estrogen-dependent signal transduction inhibition [5].

A key molecular mechanism of endocrine resistance in HR+ breast cancer involves the dysregulation of the cyclin D–CDK4/6–Rb signaling axis which controls the transition from the G1 to S phase of the cell cycle. Activating this pathway leads to retinoblastoma protein (pRb) phosphorylation, allowing for the release of E2F transcription factors and the expression of genes crucial for DNA replication and cell cycle progression [6]. Such dysregulation is often due to CCND1 gene amplification, which encodes cyclin D1, and/or increased cyclin-dependent kinase 4 and 6 (CDK4/6) expression, which allows tumor cells to bypass the antiproliferative effects of hormonal therapy [7]. Introducing cyclin-dependent kinase 4 and 6 inhibitors (CDK4/6i) has changed therapeutic paradigms, as their synergistic use with endocrine therapy significantly prolongs progression-free survival (PFS) and effectively mitigates clinically relevant endocrine resistance [8] in this patient population compared to ET alone.

The most important aspect of endocrine resistance to the standard first line endocrine therapy is a mutation of the ligand-binding domain (LBD) of Estrogen Receptor 1 (ESR1) encoding estrogen receptor α (ER). Today, it is known that the ESR1 mutations occur with a wide-ranging prevalence (10–50%) in metastatic BC (mBC) and represents the major key mechanism of endocrine resistance to the standard first line endocrine therapy.

These genomic alterations are mostly missense mutations clustered in codons 537 and 538 of the LBD are key mechanism for acquired resistance to endocrine therapy (ET) in ER-positive breast cancer, causing the estrogen receptor (ER) to stay active even without estrogen, driving tumor growth and are mainly detectable in cases of endocrine resistance after exposition to aromatase inhibitors. ESR1 mutations are present in less than 3% of mBC patients at disease onset. Most common ESR1 mutations are Y537S and cover 85% of ESR1 gene mutations. These alteration often emerge under the selective pressure of prolonged ET, leading to ligand-independent ER signaling and have been associated with poor clinical outcome [9].

Ribociclib (LEE011, Kisqali^®^) is a highly selective, orally bioavailable CDK4/6i, which induces cell cycle arrest in the G1 phase by blocking retinoblastoma (pRb) phosphorylation. CDK4/6 inhibition by means of this molecule may additionally modulate signaling pathways associated with endocrine resistance development, thereby potentially prolonging or overcoming therapeutic refractoriness induced by aberrant CDK4/6 axis activation [2,10].

The clinical efficacy of ribociclib was confirmed in three pivotal phase III studies—MONALEESA-2, MONALEESA-3, and MONALEESA-7—which served as the basis for its regulatory approval and positioning as a first-line therapy of choice (NCCN Category 1) for treating HR+/HER2− metastatic breast cancer. In the aforementioned trials, the combined use of ribociclib and endocrine therapy resulted in a statistically significant prolongation of PFS, with a reduction in the risk of progression or death ranging from 41 to 45% compared with endocrine therapy alone (median follow-up: 19.2–26.4 months) [11,12,13]. Furthermore, in all three key subpopulations—pre-, peri-, and postmenopausal patients—a significant overall survival (OS) benefit was noted, with risk of death reduced to between 24 and 27% during long-term follow-up of 4.5 to 6.6 years [14,15,16]. To date, three CDK4/6 inhibitors have been approved for use in the first- and second-line HR+/HER2-negative metastatic breast carcinoma [17,18,19].

Data from real clinical practice represent very useful insights, especially in patient populations often excluded from clinical studies, such as those with comorbidities and poor performance status [20].

The aim of our study was to evaluate patients’ clinical characteristics; the clinical effectiveness of treatment, measured by progression-free survival (PFS); and the safety profile of the combined ribociclib (CDK4/6i) and standard endocrine therapy (aromatase inhibitor) as a first-line treatment for patients with HR+/HER2− advanced or metastatic breast cancer.

## 2. Materials and Methods

### 2.1. Patient Population

In this study, we present a retrospective observational analysis of all patients with HR+/HER2− metastatic breast cancer treated with a combination of ribociclib and aromatase inhibitors in the first-line treatment of metastatic HR+/HER2− BC between June 2022 and January 2025, with a follow-up completed in October 2025. Data were collected from the Heliant electronic database and patient medical records and this study was approved by the Ethics Committee of the University Clinical Center in Niš.

A total of 132 patients who met the following criteria were included: all were older than 18 years and had pathologically confirmed HR-positive, HER2-negative breast cancer. Hormone receptor status was defined according to the immunohistochemical staining degree, and all patients with estrogen receptor (ER) or progesterone receptor (PR) expression greater than 10% were considered candidates for endocrine therapy. HER2 status was defined as an IHC score of 0 or 1+, and in cases scoring 2+, additional FISH analysis was performed to confirm that no gene amplification was present. Metastatic disease was confirmed either by imaging methods or by biopsy of a metastatic site. Ribociclib was prescribed at a dose of 600 mg per day for 21 days, followed by 7 days off, while aromatase inhibitors (letrozole 2.5 mg or anastrozole 1 mg) were prescribed daily. In cases of dose reduction, ribociclib was prescribed at a dose of either 400 mg or 200 mg for 21 days, followed by 7 days off. In premenopausal patients, it was mandatory to use LHRH agonists or laparoscopic ovariectomy before starting therapy.

Treatment response evaluation was performed every 12 weeks according to current radiological RECIST 1.1 criteria.

Progression-free survival (PFS) was defined as the time from initiating ribociclib to its discontinuation due to disease progression, or death from any cause. The objective response rate (ORR) was defined as the percentage of patients who achieved complete response (CR) and partial response (PR), while the clinical benefit rate (CBR) was defined as the percentage of patients with CR, PR, and stable disease (SD).

Adverse effects were recorded at each patient visit and graded according to the Common Terminology Criteria for Adverse Events (CTCAE) Version 5.0.

Endocrine resistance was defined as relapse during adjuvant endocrine therapy (ET) or one year after finishing. It can be primary defined as relapse within first 2 years of adjuvant endocrine therapy and secondary relapse after first 2 years of ET or 1 year after finishing adjuvant ET.

### 2.2. Statistical Analysis

Descriptive statistics were used to summarize patient characteristics. Continuously scaled measures were summarized by median values, while categorical data, including frequencies and percentages, are described in tables.

The primary endpoint was progression-free survival (PFS), defined as the time from initiating CDK4/6 inhibitor therapy until the first documented disease progression, death from any cause, whichever occurred first. Kaplan–Meier analysis and Cox Proportional Hazard Ratio were used to analyze progression-free survival (PFS), with a *p*-value of *p* < 0.05 considered statistically significant. The MedCalc software version 23.4.5 was used for statistical processing.

## 3. Results

A total of 132 female patients with a mean age of 63.5 years were included in this study, with most being postmenopausal (119; 90.1%) and 12 (9.1%) premenopausal. The analyzed group of patients also included one male. The patients had a good performance status: 72 (54.5%) were ECOG 0 and 58 (44%) were ECOG 1. Ductal invasive breast cancer was confirmed in 67 (50.8%) patients from our group, while 93 (70.5%) had grade II tumors. Dissemination to regional lymph nodes was found in 89 patients: 44 (33.3%) had N1, 26 (19.7%) had stage N2, and 19 (14.4%) had stage N3 disease.

Two patients had a slightly worse ECOG 2 performance status and neoadjuvant therapy was administrated in 31 patients (23.5%). Other characteristics are shown in Table 1.

Adjuvant chemotherapy was administrated in 52 patients (39.4%), de novo metastatic disease was present in 45 (34%), and chemotherapy (CT) for metastatic disease was administered in 24 (18.2%). From 52 patients who received adjuvant chemotherapy, 21 received antracyclines only, 27 anracycline/taxanes sequence and 4 taxanes only. A mean number of nine CT cycles was received in this subgroup, while as many as 108 patients did not receive CT for metastatic disease.

Bone-only metastases were observed in 55 patients (41.7%), while in 77 (58.3%), the metastatic process was present in two or more organs, including the liver, lungs, lymph nodes, skin, and central nervous system. Metastatic changes in the liver were present in 28 patients, while 36 patients had changes in the lungs, 25 had changes in the lymph nodes, and 3 had changes in the CNS system. Additional characteristics can be seen in Table 2.

Response rate (RR) and progression-free survival (PFS): the overall response rate (ORR) was 41.7%, while the clinical benefit rate (CBR) was 89.3% (Table 3).

**Figure 1:**
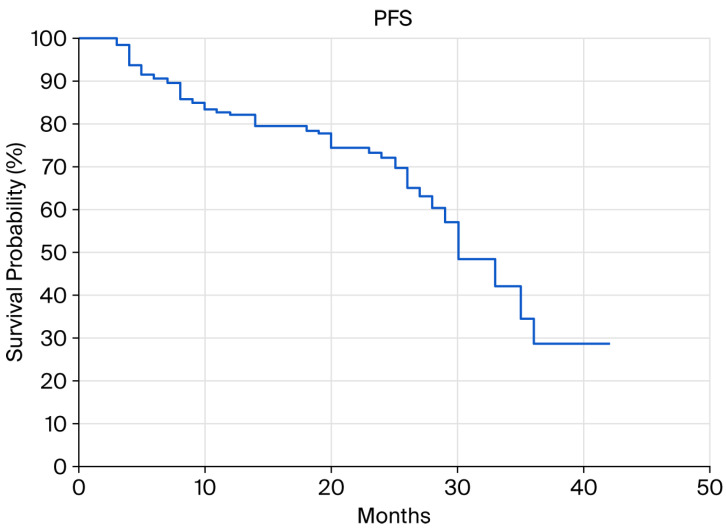
Progression-free survival (PFS).

The median progression-free survival (PFS) in the entire group was 30 months, while the 12-, 24-, and 36-month PFS values were 82.15%, 72.24%, and 28.75%, respectively (Figure 1).

**Figure 2:**
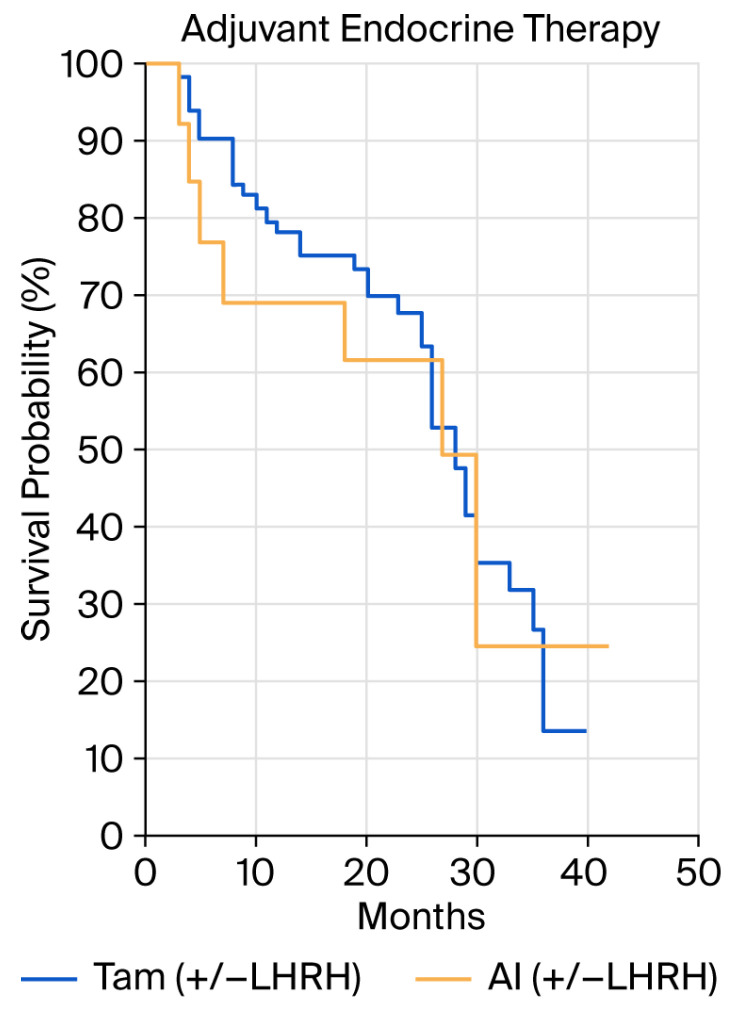
Progression-free survival (PFS) by type of adjuvant endocrine therapy (tamoxifen +/− LHRH vs. aromatase inhibitors +/− LHRH).

There was no statistically significant difference in PFS with respect to tumor grade (*p* = 0.54, grade 1 vs. grade 2 HR 0.71 (95%CI 0.29–1.71); grade 1 vs. grade 3 HR 0.56 (95%CI 0.20–1.56); grade 2 vs. grade 3 HR 0.78 (95%CI 0.40–1.56)), Ki 67 level (<20% vs. >20%, *p* = 0.83; HR 0.94, 95%CI 0.52–1.70), or type of adjuvant endocrine therapy used (tamoxifen vs. AI, *p* = 0.84; HR 0.92 (95%CI 0.39–2.16)) (Figure 2).

**Figure 3:**
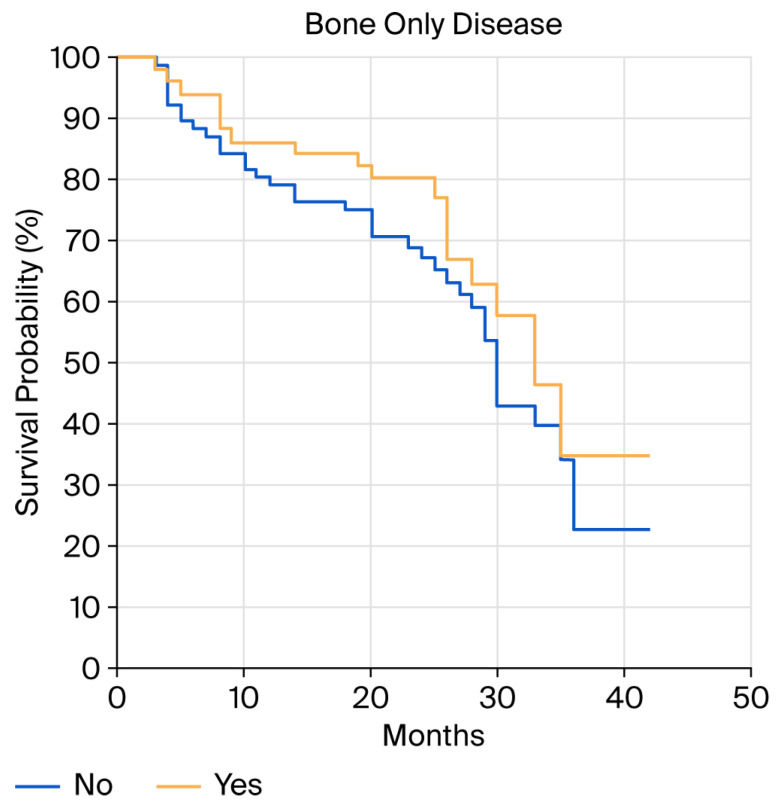
Progression-free survival (PFS) in bone-only versus not-bone-only disease.

Although a numerical difference in PFS was found in patients with bone-only metastases compared to those with metastases in other organs, the difference was not statistically significant (PFS of 33 vs. 30 months, *p* = 0.27; HR 1.35 (95%CI 0.78–2.33) Figure 3). Efficacy was consistent across menopausal status groups (menopausal PFS of 30 months vs. premenopausal PFS of 29 months, *p* = 0.81; HR 1.13 (95%CI 0.42–3.01)).

There was also no difference in PFS between endocrine-sensitive and -resistant patient populations (*p* = 0.91; HR 1.03 (95%CI 0.57–1.87)).

**Figure 4:**
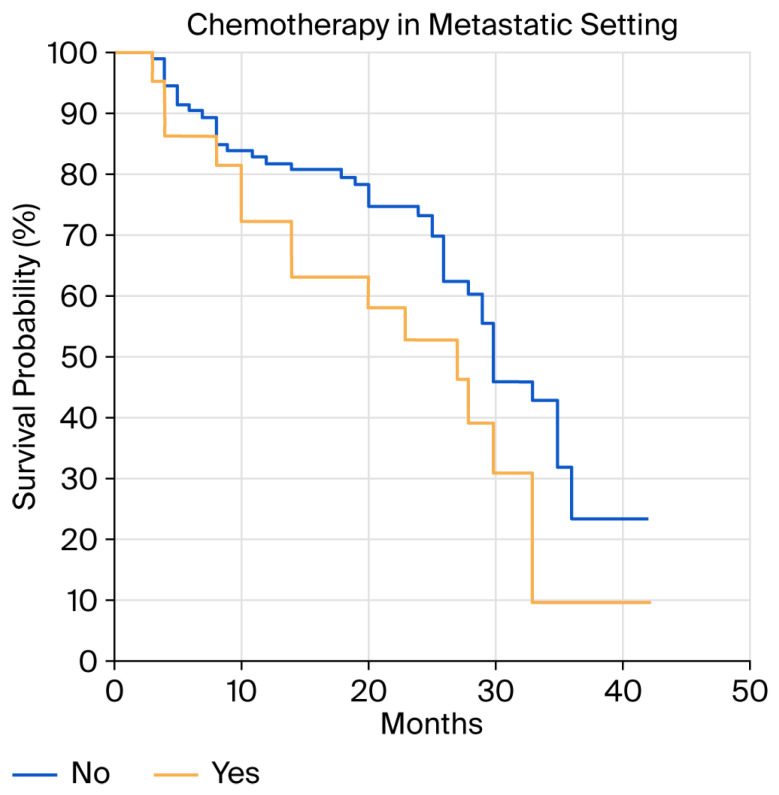
Progression-free survival (PFS) in patients who received chemotherapy for metastatic disease versus patients who did not.

It is important to emphasize that female patients who had not previously received chemotherapy had a better response to ribociclib compared to those who had (33 m vs. 28 m, *p* = 0.05, HR 0.51 (95%CI 0.2551–1.0398) Figure 4).

### Adverse Effects of Therapy

The most common adverse effect was neutropenia, occurring in 89.4% of patients, 47.7% of whom presented with grade 3 or 4. As for hepatotoxicity, a transaminase increase occurred in 25 patients (18.8%), 5 of whom (3.8%) were grade 3/4. QTc interval prolongation occurred in 5.3% of patients.

The most common side effects are shown in Table 4, while dose reduction rates are shown in Table 5.

In the evaluated patient population, 89 received the full dose of ribociclib (600 mg per day), while for the remaining patients, this was reduced due to adverse events: 32 received 400 mg of ribociclib per day (first-level dose reduction), and 5 received 200 mg per day (second-level dose reduction). Due to poor tolerance of ribociclib, a switch was made in six patients to another CDK4/6 inhibitor (palbociclib).

## 4. Discussion

Over the past decade, significant progress has been made in treating HR+/HER2-negative mBC through introducing CDK 4/6 inhibitors into clinical practice. For the first time, the median progression-free survival in first-line therapy now exceeds 2 years. The results of our study confirmed the efficacy and acceptable toxicity of combining ribociclib with aromatase inhibitors for the first-line treatment of HR+/HER2-negative mBC. The 12-month PFS interval in our study was 82.15%, while in the MONALEESA-2 and MONALEESA-7 studies it was slightly lower (74.53% and 72.7%, respectively) [2,11,13,14].

The median age in our patient group was 63.5, similar to the 62 years in the MONALEESA-2 study, and in our study, a total of 45 patients (34%) presented with de novo mBC, which is identical to the rate of 34.1% in MONALEESA-2. Most patients (50.7%) received adjuvant treatment with tamoxifen, while this number was slightly lower (41.9%) in the registration study.

Regarding metastatic disease presentation, 41.7% of patients had one metastatic site, which was slightly more than the 29.9% in MONALEESA-2. A total of 33.6% had visceral metastases (14.7% in the liver and 18.9% in the lung), lower than the 59% in MONALEESA-2. As for the patient population, it was observed that our patient group had less aggressive disease compared to the MONALEESA-2 study [11].

The response rate to therapy was similar to that in MONALEESA-2 (41.7% vs. 42.5%), but the clinical benefit rate was slightly higher (89.3% vs. 80.1%) [10,20]. The median PFS in our analysis was an impressive 30 months, slightly longer than the 25.3 in the ITT population and the 27.6 in the European Union patient population from MONALEESA-2 [11]. The benefit was consistent across all examined subgroups, regardless of tumor grade, Ki67 value, bone-only disease vs. other metastases, menopausal status, and endocrine sensitivity.

Patients without metastatic disease who had not previously been treated with chemotherapy had a 5-month-longer PFS (33 vs. 28 months), which was both clinically and statistically significant.

The pooled analysis of the MONALEESA-2, -3, and -7 studies, aiming to evaluate the efficacy of ribociclib combined with endocrine therapy in patients aged <65, 65–74, and ≥75 years, showed a similar PFS interval to that in our study (31.8, 35.7, and 31.1 months, respectively), demonstrating efficacy in all subgroups regardless of age, and confirming that the combination of ribociclib and endocrine therapy should be the treatment of choice in both younger and older patients [21]. Owing to these results, ribociclib has the highest score on the ESMO MCBS scale [22].

Premenopausal patients represented 9.1% of our patient group, and their PFS lengths were found to be consistent with those of menopausal patients.

In the real-world RIBANNA study, a slightly greater benefit was noted in the patients older than 75 years compared to those younger than 75 years, which indicates good tolerability with excellent efficacy in the older patient population (HR, 0.756; 95%CI 0.594–0.962). To date, ribociclib is the only CDK4/6 inhibitor with an overall survival benefit in this patient population [23].

As for the adverse effects of therapy, it is very important that clinicians be proficient in early recognition, as well as in managing drug dose reduction. A well-established protocol includes two levels of reduction, first to a 400 mg and second to a 200 mg daily dose. Although all three CDK4/6 inhibitors have a similar toxic drug profile, each has its own specificity, such as hepatotoxicity and QtC prolongation in ribociclib or diarrhea in abemaciclib [24,25,26].

In our study, neutropenia was the most common hematological toxicity, occurring in 89.4% of patients, 47.7% of whom exhibited grade 3 or 4 neutropenia. In the pooled analysis of MONALEESA-2, -3, and -7, grade 3 or 4 neutropenia occurred in as many as 61% of patients (52% grade 3 and 9% grade 4). Our patient population exhibited a higher incidence (28.9%) of any-grade anemia, with grade 3 or 4 anemia in 5.3% compared to less than 4% in the pooled MONALEESA. In our study, QT interval prolongation occurred at a rate of only 5.3% (grade 3–4 in 0.8%), similar to the 6.5% (grade 3–4 in 1.2%) in the pooled analysis. Transaminase spikes were confirmed in 18.8% of patients (a higher grade in 3.8%), compared to 8% in the pooled analysis. Rash occurred far less frequently in our patients—9% compared to 18% in the pooled population. Dose reductions and treatment switches occurred in 32.6% of patients, lower than the 42.5% reported in MONALEESA [26]

The pooled analysis of MONALEESA-2, -3, and -7 demonstrated ORR and CBR comparable to our results (ORR 47.5 vs. 41.7 and CBR 87.6% vs. 89.3%) in patients treated with first-line ribociclib [26].

The recently published final results of the AMALEE randomized phase 2 study, an open-label, interventional, non-inferiority study which included newly diagnosed patients with HR+/HER2− mBC and randomized them to receive either 400 mg or 600 mg of ribociclib in combination with Ais, confirmed 600 mg of ribociclib to be the appropriate starting dose in mBC while supporting dose reduction to manage dose-dependent AEs [27].

Considering the long-standing use of ribociclib in real clinical practice, lots of data have been collected from various countries. In 2025, Singer CF et al. published the results of a study including 281 patients from Austria treated with first-line ribociclib for HR+/HER2-negative mBC with a median PFS of 27.8 months, similar to our study. Dose reduction was recorded in 50.9% of patients, and a slightly greater number of patients experienced grade ≥ 3 hepatotoxicity (8.5%) [28].

In the largest comparative study in the U.S, P-VERIFY, which used the FLATIRON database for all three CDK 4/6 inhibitors, the median PFS in the patients treated with ribociclib in combination with AI was 22.9 months. This study’s results showed no statistically significant difference in PFS among all three CDK4/6 inhibitors combined with AI [29,30]. Similar results were obtained in the OPAL registry in Germany (N = 623), where no significant difference was found in rwPFS between palbociclib and ribociclib combined with ET (aHR 1.01, 95%CI 0.80–1.27) [31].

The RIBANNA prospective, non-interventional, multi-center study conducted in Germany primarily evaluated the efficacy of ribociclib with AI or fulvestrant in pre-/perimenopausal and postmenopausal women. The median PFS in the entire population was an impressive 35 months, but the results are not fully comparable with ours due to the difference in the patient population and because ET included both AI and fulvestrant [32].

Another recently published study, PALMARES, compared the effectiveness of first-line palbociclib, ribociclib, and abemaciclib in combination with ET in HR-positive/HER2-negative aBC across 18 Italian centers. The primary endpoint of this analysis was real-world progression-free survival (rwPFS). Out of 1982 patients, 736 were treated with ribociclib. Median rwPFS and OS in the whole study cohort were 34.1 months (IQR 13.9–86.3 months) and 65.9 months (IQR 36.7 months—not reached), respectively. Both abemaciclib and ribociclib had a better rwPFS compared to palbociclib (abemaciclib vs. palbociclib: aHR 0.76, 95%CI 0.63–0.92, *p* = 0.004; ribociclib vs. palbociclib: aHR 0.83, 95%CI 0.73–0.95, *p* = 0.007) [33].

In the BrasiLEEira study, which included 76 patients from 11 Brazilian centers treated with a first-line combination of ribociclib and AI, the reported one-year PFS was 77.6%, slightly lower than in our population (82.15%). Dose reductions were required in 37.7% of patients, which is comparable to our results [34].

Our study has limitations, one being that patients with endocrine-resistant disease who had previously received adjuvant AI had to start treatment with a combination of ribociclib and AI, rather than fulvestrant, due to the regulations prescribed by the National Health Insurance Fund in Serbia. Moreover, the follow-up period was relatively short, preventing us from assessing how PFS affected the overall survival outcome.

## 5. Conclusions

Combining ribociclib and AI in the first-line treatment of HR+/HER2-negative mBC has shown remarkable efficacy with a favorable drug toxicity profile in pre- and postmenopausal women. The PFS and AE results are consistent with those of pivotal studies and real clinical practice data, but a direct comparison is not possible due to differences in patient populations.

Ribociclib once again demonstrates its efficacy in all patient subgroups and remains the gold standard, alongside ET, for first-line HR+/HER2-negative mBC.

## Figures and Tables

**Table 1:** Patient characteristics (N = 132).

Gender	Female	131	99.2
	Male	1	0.8
Menopausal status	Premenopausal	12	9.1
	Postmenopausal	119	90.1
ECOG performance status	0	72	54.5
	1	58	44
	2	2	1.5
Histology	Invasive ductal carcinoma (IDC)	67	50.8
	Ductolobular carcinoma	36	27.2
	Invasive lobular carcinoma (ILC)	23	17.4
	Mucinosum carcinoma	3	2.3
	Invasive papillary carcinoma	3	2.3
Tumor grade	Grade I	7	5.3
	Grade II	93	70.5
	Grade III	6	4.5
	Grade I–II	4	3.0
	Grade II–III	15	11.4
	Unknown	7	5.3
Proliferative Ki67 index	Ki67 < 20	46	34.8
	Ki67 > 20	67	50.8
	Unknown	19	14.4
Neoadjuvant HT	Yes	31	23.5
	No	101	76.5
Type of surgery	Radical mastectomy with ALND	71	53.8
	Breast sparing surgery with ALND	14	10.6
	Breast sparing surgery with SLNB	2	1.5
	Simplex mastectomy	2	1.5
	Nipple-sparing mastectomy	1	0.8
	No surgery	42	31.8
Tumor size	T0	1	0.8
	T1	42	31.8
	T2	65	49.2
	T3	6	4.5
	T4	17	12.9
	Unknown	1	0.8
Lymph node status	N0	42	31.8
	N1	44	33.3
	N2	26	19.7
	N3	19	14.4
	Unknown	1	0.8

ECOG: Eastern Cooperative Oncology Group; ALND: Axillary Lymph Node Dissection; SLNB: Sentinel Lymph Node Biopsy.

**Table 2:** Types of adjuvant therapy and metastatic disease presentation.

Variable	Level	Frequency	Percentage
Adjuvant CT	No	80	60.6
	Yes	52	39.4
Postoperative radiotherapy	No	88	66.7
	Yes	44	33.3
Adjuvant hormonotherapy	Tamoxifen	67	50.7
	IA	8	6.1
	Tamoxifen switch IA	5	3.8
	No one	52	39.4
Metastatic presentation	De novo/treatment-naïve	45	34
	Recurrent/previously treated	87	66
Number of metastatic sites	One metastatic site (bones)	55	41.7
	Two or more metastatic sites	77	58.3
Site of metastasis	Bones	90	47.4
	Liver	28	14.7
	Lungs	36	18.9
	Skin	8	4.2
	Lymph nodes	25	13.2
	Central nervous system (CNS)	3	1.6
CT for metastatic disease	No	108	81.8
	Yes	24	18.2

CT: chemotherapy; IA: aromatase inhibitor.

**Table 3:** Response to therapy.

Response to Therapy	132 (100%)
Stable disease	63 (47.7%)
Partial response	49 (37.1%)
Complete response	6 (4.5%)
Progression of disease	14 (10.7%)
Overall response rate (PR + CR)	55 (41.7%)
Clinical benefit rate (SD + PR + CR)	118 (89.3%)

**Table 4:** Prevalence of toxicities in patients.

Toxicity	Any Grade	Grade 1–2	Grade 3–4
Neutropenia	118 (89.4%)	55 (41.7%)	63 (47.7%)
Anemia	34 (28.9%)	27 (23.6%)	7 (5.3%)
Thrombocytopenia	12 (9.0%)	11 (8.3%)	1(0.7%)
Elevated transaminase	25 (18.8%)	20 (15%)	5 (3.8%)
Prolonged QTc interval	7 (5.3%)	6 (4.5%)	1 (0.8%)
Skin toxicity	12 (9%)	6 (6%)	4 (3%)

**Table 5:** Dose reduction and switch to another CDK 4/6i.

Variable	Level	Frequency	Percentage
Dose	Starting dose (600 mg/day)	89	67.4
	First dose reduction (400 mg/day)	32	24.2
	Second dose reduction (200 mg/day)	5	3.8
Switch to other CDK4/6i	Palbociclib	6	4.6

## Data Availability

The original contributions presented in this study are included in the article. Further inquiries can be directed to the corresponding author.
